# Emergence of GII.4 Sydney[P16]-like Norovirus-Associated Gastroenteritis, China, 2020–2022

**DOI:** 10.3201/eid2909.230383

**Published:** 2023-09

**Authors:** Yuanyun Ao, Lijuan Lu, Jin Xu

**Affiliations:** Children's Hospital of Fudan University, Shanghai, China (Y. Ao, L. Lu, J. Xu);; Shanghai Institute of Infectious Disease and Biosecurity of Fudan University, Shanghai, China (J. Xu).

**Keywords:** Norovirus, viruses, acute gastroenteritis, enteric infections, GII.4 Sydney[P16], phylogenetic analysis, China

## Abstract

Newly evolved GII.4 Sydney[P16] norovirus with multiple residue mutations, already circulating in parts of China, became predominant and caused an abrupt increase in diagnosed norovirus cases among children with gastroenteritis in Shanghai during 2021–2022. Findings highlight the need for continuous long-term monitoring for GII.4 Sydney[P16] and emergent GII.4 norovirus variants.

Norovirus, the main cause of nonbacterial acute gastroenteritis (AGE) outbreaks worldwide (*1*), is a positive-sense, single-stranded RNA virus within the family Caliciviridae. Its genome contains 3 open reading frames (ORFs): ORF1 encodes a polyprotein cleaved into 6 nonstructural proteins, including RNA-dependent RNA polymerase (RdRp); ORF2 encodes major (VP1) and ORF3 minor (VP2) capsid proteins (*2*). On the basis of VP1 amino acid sequences, noroviruses can be grouped into 10 genogroups (GI–GX) and 49 genotypes (*3*); GI and GII genogroups are the most common in human infections. 

Since 2002, GII.4 has been the predominant norovirus genotype worldwide (*4*,*5*). GII.4 Sydney norovirus recombinant with a GII.P31 polymerase, GII.4 Sydney[P31], emerged in 2012 and caused pandemic illness during 2012–2013 (*6*). However, in 2015, a recombinant GII.4 Sydney[P16] norovirus emerged and recently became predominant in some Western countries (*7*–*10*). GII.4 Sydney[P16] norovirus has advantageous epidemic potential because of viral fitness from its recombinant components: emerging GII.P16 polymerase and persistently mutating GII.4 VP1 (*11*,*12*). Although GII.4 Sydney[P16] norovirus prevalence has rarely been reported in China (*13*), its advantageous qualities raise concerns about the virus possibly causing an epidemic. To monitor epidemiologic and genetic data from GII.4 Sydney[P16] norovirus in China, we performed laboratory-based surveillance of noroviruses among children with AGE in Shanghai. 

## The Study 

Beginning in 2016, fecal specimens were collected from children ≤5 years of age with AGE seen as outpatients at Children’s Hospital of Fudan University in Shanghai; case-patients from local outbreaks were excluded. AGE is defined as 3 episodes of loose feces or 2 episodes of vomiting within 24 hours. We tested fecal samples for GI and GII norovirus by dual-genotyped reverse transcription PCR (RT-PCR), as described elsewhere (*14*). We genotyped sequences using the norovirus typing tool of the Dutch National Institute for Public Health and the Environment (https://www.rivm.nl/mpf/norovirus/typingtool). We measured concentrations of viral RNA in norovirus-positive samples using real-time RT-PCR with primers/probe targeting the conserved ORF1–ORF2 junction, as described elsewhere (*15*). 

**Figure 1:**
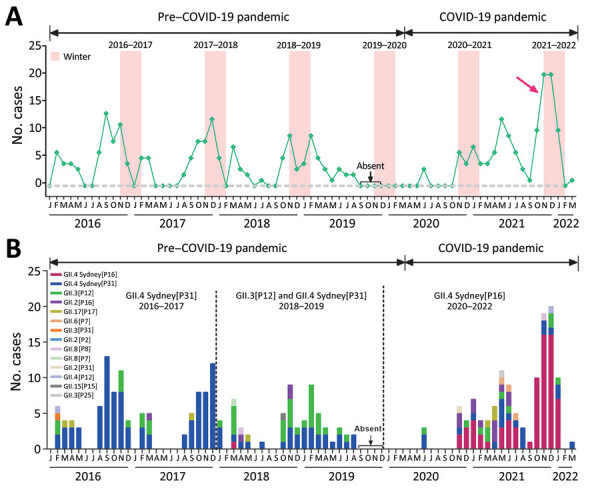
Emergence of recombinant GII.4 Sydney[P16] norovirus associated with acute gastroenteritis among children treated as outpatients in Shanghai, before and during COVID-19 pandemic, Shanghai, China, during January 2016–March 2022. A) Numbers of cases of norovirus-associated acute gastroenteritis. Red arrow indicates an abrupt increase in norovirus activity. B) Genotype (polymerase-capsid) distribution of norovirus. Different norovirus genotypes are indicated by color (key). Start date of COVID-19 pandemic declared by World Health Organization was March 11, 2020; absent labels indicate period (September–December 2019) during which fecal sample collection was paused.

We determined that, during January 2016–March 2022, a total of 301/2,419 (12.4%) fecal samples from cases in children were norovirus-positive (Figure 1, panel A). Annually, the peak number of norovirus cases has been detected in samples taken during winter, exhibiting a seasonal characteristic. Each year during 2016–2019, there were <60 norovirus cases; during 2020, the first year of the COVID-19 pandemic, norovirus activity abruptly decreased to 13 cases, but rates then unexpectedly increased to 110 cases in 2021, a trend similar to the proportion of norovirus cases among all gastroenteritis cases (data not shown). Of note, 40 (36.4%) cases were identified in samples taken during November–December 2021; a total of 11 cases were detected in samples taken during January–March 2022. 

We observed a dynamic profile of norovirus genotypes in cases among children during 2016–2022 (Figure 1, panel B). Before the COVID-19 pandemic, the predominant genotypes were GII.4 Sydney[P31] during 2016–2017 and GII.4 Sydney[P31] and GII.3[P12] during 2018–2019; however, GII.4 Sydney[P16] suddenly emerged in November 2020 and predominated in 2021. Of 110 norovirus samples in 2021, we successfully genotyped 100. GII.4 Sydney[P16], the predominant genotype, was identified in 60 (60%) samples, followed by GII.4 Sydney[P31] in 14 (14%), GII.2[P16] in 9 (9%), GII.3[P12] in 7 (7%), GII.17[P17] in 3 (3%), GII.6[P7] in 3 (3%), and other genotypes in 2 (2%). Of note, the proportion of GII.4 Sydney[P16] cases rose sharply to 42/55 (76.4%) during October–December 2021 and 7/11 (70%) cases during January–March 2022. 

**Figure 2:**
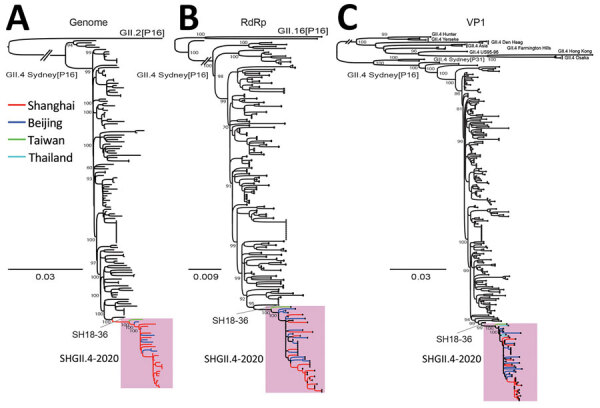
Phylogenetic analysis of newly identified GII.4 Sydney[P16] noroviruses in Shanghai, China, 2020–2022. Maximum-likelihood phylogenetic trees show complete genome (A) RNA-dependent RNA polymerase (RdRp) (B) and VP1 (C) gene sequences for newly identified GII.4 Sydney[P16] strains (n = 23) in Shanghai. Pink shading indicates new sublineage (tentatively named SHGII.4-2020) in GII.4 Sydney[P16] cluster. Branches of each strain in SHGII.4-2020 are indicated by color according to identified positions; red indicates GII.4 Sydney[P16] strains identified in this study, except SH18-36. Numbers on ancestral nodes represent node support values.

Using high-throughput sequencing, we identified 23 complete genomes of GII.4 Sydney[P16] (GenBank accession nos. OP037976–83 and OQ940068–82) from 23 case-patients. Maximum-likelihood phylogenetic trees of GII.4 Sydney[P16] full-length RdRp and VP1 genes all showed that 22 strains from November 2020–December 2021 clustered together with strains recently identified in Beijing (GenBank accession nos. OL336332.1, OL336335.1–41.1, and OL336352.1–89.1), Taiwan (ON329737.1), and Thailand (MW521126.1–7.1 and MW521129.1–30.1) (Appendix Table), which evolved into a genetically distinct sublineage (tentatively named SHGII.4-2020) in the GII.4 Sydney[P16] cluster (Figure 2). All trees showed SHGII.4-2020 most closely related to SH18-36, the first identified GII.4 Sydney[P16] strain in our study, implying that SH18-36 might constitute an evolutionary ancestor of SHGII.4-2020. 

Compared with sequences of GII.4 Sydney[P16] strains from GenBank, SHGII.4-2020 had 19 aa mutations: 10 in nonstructural proteins (T169S and L305F in p48; K84R, V88I and I168V in p22; K103R in VPg; K54R, V125A, A311T and N427T in RdRp), 2 in VP1 (R297H and D372N), and 7 in VP2 (K80R, A108V, A128S, N157T, T164A, I174T and N207S) (Appendix Figure 1). In VP1, R297H and D372N substitutions were mapped to the main antigenic epitope A, and D372N was also present around the HBGA binding site II (Appendix Figure 2). Those 2 residues, 297H and 372N, were only identified in certain previous GII.4 Sydney[P16] strains (MG002631.1, MH922876, and MK754444). Three substitutions, K54R, V125A, and N427T, were located on the surface of RdRp (Appendix Figure 2); A312T resided adjacent to motif B. In addition, we found 7 unique mutations in Shanghai strains. Further studies of these SHGII.4-2020 mutations are needed to better understand their role in its emergence. 

We performed comparative clinical analysis of SHGII.4-2020, GII.4 Sydney[P31] and GII.3[P12] cases. The median age of SHGII.4-2020 case-patients (21.5 months, interquartile range [IQR] 15–50.3 months) was older than those for GII.4 Sydney[P12] (12 months, IQR 9–19.3 months) and GII.3[P12] (12 months, IQR 8–26.75 months; p<0.005) case-patients (Table). We observed SHGII.4-2020 more commonly than GII.4 Sydney[P31] among children >36 months of age, whereas the converse was true among children <12 months of age (p<0.005) (Table). Vomiting was a more common clinical sign among SHGII.4-2020 case-patients than among GII.4 Sydney[P31] and GII.3[P12] case-patients (p<0.05) (Table). The median viral RNA load (cycle threshold value) for SHGII.4-2020 (15.86, IQR 13.95–18.74) was higher than those for GII.4 Sydney[P31] (17.00, IQR 15.11–20.32; p = 0.0372) and GII.3[P12] (17.98, IQR 15.76–21.75; p = 0.0093) (Appendix Figure 3). Five samples with high cycle threshold values (>25.0) for each genotype were randomly selected for primer/probe sequence mismatch analysis; no mismatch was found. 

**Table:** Comparisons of sociodemographic and clinical characteristics between GII.4 Sydney[P16] norovirus and GII.4 Sydney[P31]/GII.3[P12] norovirus infection in norovirus–positive children, Shanghai, China, January 2016–March 2022*

Characteristic	GII.4 Sydney[P16]	GII.4 Sydney[P31]	p value†	GII.3[P12]	p value‡
Total no. patients	63	120		47	
Median patient age, mo (IQR)	21.5 (15–50.3)	12 (9–19.3)	<0.0001§	12 (8–26.75)	0.004§
Age range, mo					
<12	9 (14.3)	47 (39.2)	**0.001**¶	18 (38.3)	**0.004**¶
12–36	31 (49.2)	63 (52.5)	0.672¶	19 (40.4)	0.360¶
>36	23 (36.5)	10 (8.3)	**<0.001**¶	10 (21.3)	0.085¶
F:M ratio	0.58:1	0.5:1	0.744#	0.48:1	0.688#
Signs/symptoms					
Total no. patients with signs/symptoms	60	112	NA	44	NA
Diarrhea	45 (75.0)	93 (83.0)	0.586¶	33 (75.0)	0.579¶
Duration <5 d	33 (73.3)	64 (68.8)	0.821¶	26 (78.8)	0.837¶
Duration >5 d	12 (26.7)	29 (31.2)	0.687¶	7 (21.2)	0.664¶
Vomiting	35 (58.3)	40 (35.7)	**<0.001**¶	17 (38.6)	**0.047**¶
Fever	18 (30.0)	20 (17.9)	0.067¶	17 (38.6)	0.357¶
Abdominal cramps	9 (15)	2 (1.8)	0.02¶	6 (13.6)	0.845¶

*Values are no. (%) patients except as indicated. Bold indicates statistical significance. NA, not applicable †Denotes comparison between GII.4 Sydney[P16] and GII.4 Sydney[P31]. ‡Indicates comparison between GII.4 Sydney[P16] and GII.3[P12]. §By χ^2^ test. ¶For the comparison of the median age between groups; calculated by Mann-Whitney U-test. #By Fisher exact test.

## Conclusions

We provide evidence that GII.4 Sydney[P16] norovirus has evolved into a new sublineage, SHGII.4-2020, that carries multiple mutations and is circulating in different regions of China. We found that SHGII.4-2020 became the predominant norovirus genotype and resulted in an abrupt increase in diagnosed cases among children treated as outpatients at a hospital in Shanghai during 2021–2022. Based on data from CaliciNet China, GII.2[P16] was identified as the dominant genotype in 2016–2020 norovirus outbreaks in China (*13*), but more recent data have not been reported. The viral load in feces for SHGII.4-2020 was higher than for GII.4 Sydney[P31] and GII.3[P12] noroviruses, suggesting the higher viral replication efficiency and transmissibility of SHGII.4-2020. However, further multivariate analyses are needed to exclude potential confounding factors, such as time interval from sign and symptom onset to sample collection, which may have biased those results. The higher proportion of case-patients experiencing vomiting during infection with SHGII.4-2020 is of particular clinical and epidemiologic interest because this symptom profile may affect how that norovirus strain spreads and cause a changes in epidemiology. Although our study was limited by a small number of cases and its single-center setting, our findings highlight the need for continuous long-term monitoring for global spread of SHGII.4-2020 and emergence of newly evolving GII.4 norovirus variants. 

Appendix Additional information about study of emergence of GII.4 Sydney[P16]-like norovirus-associated gastroenteritis in China, 2021–2022.
