# Supplementary material for: Emergence of GII.4 Sydney[P16]-like Norovirus-Associated Gastroenteritis, China, 2020–2022
Source: Emerg Infect Dis. 2023 Sep;29(9):1837–41. doi: 10.3201/eid2909.230383 (PMC10461682; doi:10.3201/eid2909.230383)
Supplement: Appendix — Additional information about study of emergence of GII.4 Sydney[P16]-like norovirus-associated gastroenteritis in China, 2021–2022. [file 23-0383-Techapp-s1.pdf]

Article DOI: https://doi.org/10.3201/eid2909.230383

EID cannot ensure accessibility for supplementary materials supplied by authors. Readers who have difficulty accessing supplementary content should contact the authors for assistance.

Emergence of GII.4 Sydney[P16]-like Norovirus-Associated Gastroenteritis, China, 2020–2022

Appendix

Appendix Table. List of full-length, RdRp and VP1 nucleotide sequences from GenBank used for phylogenetic analysis of SHGII.4-2020*

Strain name	GenBank accession numbers	Genotype	Note
Pasadena 3477	KY947550.1	GII.4 Sydney[P16]	A
CA3477	KX907727.1	GII.4 Sydney[P16]	A
NORO_227_07_01_2016	MH218685.1	GII.4 Sydney[P16]	A
NOR-2559	KY887599.1	GII.4 Sydney[P16]	A
NOR-2516	KY887602.1	GII.4 Sydney[P16]	A
NORO_226_06_01_2016	NC_039477.1	GII.4 Sydney[P16]	A
NOR-2558	KY887600.1	GII.4 Sydney[P16]	A
sNOR-2518	KY887604.1	GII.4 Sydney[P16]	A
CS0275	MK762566.1	GII.4 Sydney[P16]	A
526-2	MT344179.1	GII.4 Sydney[P16]	A
526-1	MT238666.1	GII.4 Sydney[P16]	A
NOR-2565	KY887601.1	GII.4 Sydney[P16]	A
Alameda0771	MK756033.1	GII.4 Sydney[P16]	A
Alameda0766	MK756034.1	GII.4 Sydney[P16]	A
Alameda0792	MK756037.1	GII.4 Sydney[P16]	A
Alameda0791	MK756038.1	GII.4 Sydney[P16]	A
Fresno-0182	MK073885.1	GII.4 Sydney[P16]	A
Alameda0788	MK756036.1	GII.4 Sydney[P16]	A
Olmsted0261	MK773585.1	GII.4 Sydney[P16]	A
Olmsted0258	MK773584.1	GII.4 Sydney[P16]	A
St.Croix0439	MK752936.1	GII.4 Sydney[P16]	A
Porter0184	MK762639.1	GII.4 Sydney[P16]	A
NS16-C38	KY210980.3	GII.4 Sydney[P16]	A
SanDiego0043	MK753029.1	GII.4 Sydney[P16]	A
BMH16-074	MW661254.1	GII.4 Sydney[P16]	A
Gallatin-0038	MK073891.1	GII.4 Sydney[P16]	A
Gallatin-0036	MK073890.1	GII.4 Sydney[P16]	A
CS0195	MK762564.1	GII.4 Sydney[P16]	A
CS0194	MK762563.1	GII.4 Sydney[P16]	A
CS0193	MK762562.1	GII.4 Sydney[P16]	A
CS0651	MK762568.1	GII.4 Sydney[P16]	A
Kansas0843	OL898514.1	GII.4 Sydney[P16]	A
NS17-A869	MG892929.3	GII.4 Sydney[P16]	A
BaltimoreCity0391	MK754442.1	GII.4 Sydney[P16]	A
Stafford0184	MK764018.1	GII.4 Sydney[P16]	A
Marin0375	MK753030.1	GII.4 Sydney[P16]	A
Wayne0078	MK764013.1	GII.4 Sydney[P16]	A
Ingham0228	MK764015.1	GII.4 Sydney[P16]	A
Roscommon-0105	MK073888.1	GII.4 Sydney[P16]	A
Nashville-0085	MK073894.1	GII.4 Sydney[P16]	A
526-3	MT367328.1	GII.4 Sydney[P16]	A
259-3	MT238672.1	GII.4 Sydney[P16]	A
259-1	MT238670.1	GII.4 Sydney[P16]	A
259-2	MT238671.1	GII.4 Sydney[P16]	A
782-3	MT238668.1	GII.4 Sydney[P16]	A
782-1	MT238667.1	GII.4 Sydney[P16]	A

Strain name	GenBank accession numbers	Genotype	Note
259-1	MT537605.1	GII.4 Sydney[P16]	A
259-3	MT537634.1	GII.4 Sydney[P16]	A
259-2	MT537941.1	GII.4 Sydney[P16]	A
259-2	MT537633.1	GII.4 Sydney[P16]	A
782-2	MT344180.1	GII.4 Sydney[P16]	A
Miami0181	MK762638.1	GII.4 Sydney[P16]	A
LaPorte0381	MK762637.1	GII.4 Sydney[P16]	A
Marathon0626	MK752942.1	GII.4 Sydney[P16]	A
Auglaize0259	MK629457.1	GII.4 Sydney[P16]	A
G11_6	MZ478140.1	GII.4 Sydney[P16]	A
G9_4.5	MZ478139.1	GII.4 Sydney[P16]	A
G4_11	MZ478136.1	GII.4 Sydney[P16]	A
NOR-2520	KY887605.1	GII.4 Sydney[P16]	A
CS0276	MK762567.1	GII.4 Sydney[P16]	A
CS4243	KY947549.1	GII.4 Sydney[P16]	A
Alameda0787	MK756035.1	GII.4 Sydney[P16]	A
Hardin1028	MK762746.1	GII.4 Sydney[P16]	A
55HBO	MW305548.1	GII.4 Sydney[P16]	A
211HBO	MW305557.1	GII.4 Sydney[P16]	A
278HBO	MW305560.1	GII.4 Sydney[P16]	A
249HBO	MW305558.1	GII.4 Sydney[P16]	A
170HBO	MW305553.1	GII.4 Sydney[P16]	A
Jackson4273	MK762624.1	GII.4 Sydney[P16]	A
G19_10	MZ478141.1	GII.4 Sydney[P16]	A
Wood0352	MK775032.1	GII.4 Sydney[P16]	A
BNE1	MG002630.1	GII.4 Sydney[P16]	A
Lee0113	MK762629.1	GII.4 Sydney[P16]	A
LosAngeles0190	MK762635.1	GII.4 Sydney[P16]	A
Brevard0199	MT031761.1	GII.4 Sydney[P16]	A
Ramsey0269	MK773586.1	GII.4 Sydney[P16]	A
Washington0207	MK754446.1	GII.4 Sydney[P16]	A
Shawano0193	MN897756.1	GII.4 Sydney[P16]	A
QLDB309	KY905335.1	GII.4 Sydney[P16]	A
Nizhny_Novgorod2275	MZ958411.1	GII.4 Sydney[P16]	A
LaCrosse0643	MK752943.1	GII.4 Sydney[P16]	A
Lane0371	MK753017.1	GII.4 Sydney[P16]	A
CS1010	MK762570.1	GII.4 Sydney[P16]	A
CS1009	MK762569.1	GII.4 Sydney[P16]	A
ST104	MH791994.1	GII.4 Sydney[P16]	A
BMH18-085	MW661256.1	GII.4 Sydney[P16]	A
11617	MN782359.1	GII.4 Sydney[P16]	A
Natrona0468	MK762632.1	GII.4 Sydney[P16]	A
BMH19-090	MW661258.1	GII.4 Sydney[P16]	A
Loudoun0411	MK764021.1	GII.4 Sydney[P16]	A
SanJoaquin0116	MK753032.1	GII.4 Sydney[P16]	A
CerroGordo0434	MK752937.1	GII.4 Sydney[P16]	A
Brown0441	MK752938.1	GII.4 Sydney[P16]	A
Kansas11006	OL898512.1	GII.4 Sydney[P16]	A
Jackson1608	MK762621.1	GII.4 Sydney[P16]	A
Clackamas0373	MK753018.1	GII.4 Sydney[P16]	A
Grant0015	MK752934.1	GII.4 Sydney[P16]	A
Grant0016	MK752933.1	GII.4 Sydney[P16]	A
JH29	MN525276.1	GII.4 Sydney[P16]	A
JH28	MN525275.1	GII.4 Sydney[P16]	A
LosAngeles0196	MK762636.1	GII.4 Sydney[P16]	A
Baltimore0201	MK754444.1	GII.4 Sydney[P16]	A
CMH-N010-18	MW305723.1	GII.4 Sydney[P16]	A
BMH19-132	MW661282.1	GII.4 Sydney[P16]	A
BMH19-127	MW661278.1	GII.4 Sydney[P16]	A
BMH19-129	MW661280.1	GII.4 Sydney[P16]	A
BMH19-128	MW661279.1	GII.4 Sydney[P16]	A
Buchanan0126	MT032004.1	GII.4 Sydney[P16]	A
BMH19-092	MW661259.1	GII.4 Sydney[P16]	A
BMH19-093	MW661260.1	GII.4 Sydney[P16]	A
Helena0207	MT029316.1	GII.4 Sydney[P16]	A
Kansas1621	OL898515.1	GII.4 Sydney[P16]	A
NS18-A1494	MK483909.2	GII.4 Sydney[P16]	A
CMH-N033-18	MW305708.1	GII.4 Sydney[P16]	A
SCD1062-2	MN996299.1	GII.4 Sydney[P16]	A

Strain name	GenBank accession numbers	Genotype	Note
SCD1055-1	MN996297.1	GII.4 Sydney[P16]	A
1915Y216	OL336386.1	GII.4 Sydney[P16]	A
19121200	OL336382.1	GII.4 Sydney[P16]	A
21161049	OL336388.1	GII.4 Sydney[P16]	A
20062001	OL336383.1	GII.4 Sydney[P16]	A
20153072	OL336387.1	GII.4 Sydney[P16]	A
21063054	OL336385.1	GII.4 Sydney[P16]	A
20051046	OL336384.1	GII.4 Sydney[P16]	A
21051018	OL336389.1	GII.4 Sydney[P16]	A
Unknown	ON329737.1	GII.4 Sydney[P16]	A
Davidson0138	MT031821.1	GII.4 Sydney[P16]	A
Polk0185	MN897755.1	GII.4 Sydney[P16]	A
BMH19-094	MW661261.1	GII.4 Sydney[P16]	A
BNE4	MG002633.1	GII.4 Sydney[P16]	A
BMH19-137	MW661283.1	GII.4 Sydney[P16]	A
Bay0244	MK764016.1	GII.4 Sydney[P16]	A
BNE2	MG002631.1	GII.4 Sydney[P16]	A
BJSMQ	KY421122.1	GII.2[P16]	A
Pasadena 3477	KY947550.1	GII.4 Sydney[P16]	B
CA3477	KX907727.1	GII.4 Sydney[P16]	B
NORO_227_07_01_2016	MH218685.1	GII.4 Sydney[P16]	B
NOR-2559	KY887599.1	GII.4 Sydney[P16]	B
NORO_226_06_01_2016	NC_039477.1	GII.4 Sydney[P16]	B
NOR-2558	KY887600.1	GII.4 Sydney[P16]	B
LaPorte0381	MK762637.1	GII.4 Sydney[P16]	B
Marathon0626	MK752942.1	GII.4 Sydney[P16]	B
G11_6	MZ478140.1	GII.4 Sydney[P16]	B
G9_4.5	MZ478139.1	GII.4 Sydney[P16]	B
G4_11	MZ478136.1	GII.4 Sydney[P16]	B
Auglaize0259	MK629457.1	GII.4 Sydney[P16]	B
CS0275	MK762566.1	GII.4 Sydney[P16]	B
Alameda0766	MK756034.1	GII.4 Sydney[P16]	B
NOR-2565	KY887601.1	GII.4 Sydney[P16]	B
526-2	MT344179.1	GII.4 Sydney[P16]	B
Alameda0791	MK756038.1	GII.4 Sydney[P16]	B
526-1	MT238666.1	GII.4 Sydney[P16]	B
Alameda0771	MK756033.1	GII.4 Sydney[P16]	B
Fresno-0182	MK073885.1	GII.4 Sydney[P16]	B
Alameda0792	MK756037.1	GII.4 Sydney[P16]	B
Alameda0788	MK756036.1	GII.4 Sydney[P16]	B
NS16-C38	KY210980.3	GII.4 Sydney[P16]	B
NS17-A869	MG892929.3	GII.4 Sydney[P16]	B
SanDiego0043	MK753029.1	GII.4 Sydney[P16]	B
BMH16-074	MW661254.1	GII.4 Sydney[P16]	B
NOR-2516	KY887602.1	GII.4 Sydney[P16]	B
Marin0375	MK753030.1	GII.4 Sydney[P16]	B
BaltimoreCity0391	MK754442.1	GII.4 Sydney[P16]	B
Stafford0184	MK764018.1	GII.4 Sydney[P16]	B
Gallatin-0038	MK073891.1	GII.4 Sydney[P16]	B
Gallatin-0036	MK073890.1	GII.4 Sydney[P16]	B
CS0195	MK762564.1	GII.4 Sydney[P16]	B
CS0194	MK762563.1	GII.4 Sydney[P16]	B
CS0193	MK762562.1	GII.4 Sydney[P16]	B
CS0651	MK762568.1	GII.4 Sydney[P16]	B
Kansas0843	OL898514.1	GII.4 Sydney[P16]	B
NS17-A843	MG892946.3	GII.4 Sydney[P16]	B
Olmsted0261	MK773585.1	GII.4 Sydney[P16]	B
Olmsted0258	MK773584.1	GII.4 Sydney[P16]	B
St.Croix0439	MK752936.1	GII.4 Sydney[P16]	B
NOR-2518	KY887604.1	GII.4 Sydney[P16]	B
CS0276	MK762567.1	GII.4 Sydney[P16]	B
CS4243	KY947549.1	GII.4 Sydney[P16]	B
278HBO	MW305560.1	GII.4 Sydney[P16]	B
Alameda0787	MK756035.1	GII.4 Sydney[P16]	B
Hardin1028	MK762746.1	GII.4 Sydney[P16]	B
249HBO	MW305558.1	GII.4 Sydney[P16]	B
170HBO	MW305553.1	GII.4 Sydney[P16]	B
55HBO	MW305548.1	GII.4 Sydney[P16]	B
211HBO	MW305557.1	GII.4 Sydney[P16]	B

Strain name	GenBank accession numbers	Genotype	Note
Maricopa0357	MK753033.1	GII.4 Sydney[P16]	B
CS1010	MK762570.1	GII.4 Sydney[P16]	B
CS1009	MK762569.1	GII.4 Sydney[P16]	B
ST104	MH791994.1	GII.4 Sydney[P16]	B
BMH18-085	MW661256.1	GII.4 Sydney[P16]	B
11617	MN782359.1	GII.4 Sydney[P16]	B
BMH19-090	MW661258.1	GII.4 Sydney[P16]	B
Natrona0468	MK762632.1	GII.4 Sydney[P16]	B
LaCrosse0643	MK752943.1	GII.4 Sydney[P16]	B
Loudoun0411	MK764021.1	GII.4 Sydney[P16]	B
LosAngeles0196	MK762636.1	GII.4 Sydney[P16]	B
Wayne0078	MK764013.1	GII.4 Sydney[P16]	B
Ingham0228	MK764015.1	GII.4 Sydney[P16]	B
Miami0181	MK762638.1	GII.4 Sydney[P16]	B
Roscommon-0105	MK073888.1	GII.4 Sydney[P16]	B
Nashville-0085	MK073894.1	GII.4 Sydney[P16]	B
NOR-2520	KY887605.1	GII.4 Sydney[P16]	B
Porter0184	MK762639.1	GII.4 Sydney[P16]	B
NS18-A1393	MK881905.1	GII.4 Sydney[P16]	B
SanJoaquin0116	MK753032.1	GII.4 Sydney[P16]	B
CerroGordo0434	MK752937.1	GII.4 Sydney[P16]	B
Brown0441	MK752938.1	GII.4 Sydney[P16]	B
Kansas11006	OL898512.1	GII.4 Sydney[P16]	B
Jackson1608	MK762621.1	GII.4 Sydney[P16]	B
Lane0371	MK753017.1	GII.4 Sydney[P16]	B
JH29	MN525276.1	GII.4 Sydney[P16]	B
JH28	MN525275.1	GII.4 Sydney[P16]	B
NS18-A1494	MK483909.2	GII.4 Sydney[P16]	B
CMH-N033-18	MW305708.1	GII.4 Sydney[P16]	B
SCD1062-2	MN996299.1	GII.4 Sydney[P16]	B
SCD1055-1	MN996297.1	GII.4 Sydney[P16]	B
NS17-A875	MG892930.2	GII.4 Sydney[P16]	B
BMH19-132	MW661282.1	GII.4 Sydney[P16]	B
BMH19-129	MW661280.1	GII.4 Sydney[P16]	B
BMH19-128	MW661279.1	GII.4 Sydney[P16]	B
BMH19-127	MW661278.1	GII.4 Sydney[P16]	B
Buchanan0126	MT032004.1	GII.4 Sydney[P16]	B
BMH19-092	MW661259.1	GII.4 Sydney[P16]	B
BMH19-093	MW661260.1	GII.4 Sydney[P16]	B
Helena0207	MT029316.1	GII.4 Sydney[P16]	B
Nizhny_Novgorod2275	MZ958411.1	GII.4 Sydney[P16]	B
Baltimore0201	MK754444.1	GII.4 Sydney[P16]	B
GT-1	MN220720.1	GII.4 Sydney[P16]	B
QLDB309	KY905335.1	GII.4 Sydney[P16]	B
G19_10	MZ478141.1	GII.4 Sydney[P16]	B
LosAngeles0190	MK762635.1	GII.4 Sydney[P16]	B
Lee0113	MK762629.1	GII.4 Sydney[P16]	B
Wood0352	MK775032.1	GII.4 Sydney[P16]	B
Brevard0199	MT031761.1	GII.4 Sydney[P16]	B
BNE1	MG002630.1	GII.4 Sydney[P16]	B
Ramsey0269	MK773586.1	GII.4 Sydney[P16]	B
Washington0207	MK754446.1	GII.4 Sydney[P16]	B
Grant0015	MK752934.1	GII.4 Sydney[P16]	B
Grant0016	MK752933.1	GII.4 Sydney[P16]	B
BMH19-094	MW661261.1	GII.4 Sydney[P16]	B
Shawano0193	MN897756.1	GII.4 Sydney[P16]	B
Clackamas0373	MK753018.1	GII.4 Sydney[P16]	B
1915Y216	OL336386.1	GII.4 Sydney[P16]	B
19121200	OL336382.1	GII.4 Sydney[P16]	B
1908Y813	OL336378.1	GII.4 Sydney[P16]	B
20161008	OL336381.1	GII.4 Sydney[P16]	B
20051042	OL336372.1	GII.4 Sydney[P16]	B
21161049	OL336388.1	GII.4 Sydney[P16]	B
2105Y1922	OL336371.1	GII.4 Sydney[P16]	B
2105Y1920	OL336370.1	GII.4 Sydney[P16]	B
2105y1919	OL336369.1	GII.4 Sydney[P16]	B
2105Y1889	OL336368.1	GII.4 Sydney[P16]	B
20051046	OL336384.1	GII.4 Sydney[P16]	B
2005Y130	OL336379.1	GII.4 Sydney[P16]	B

Strain name	GenBank accession numbers	Genotype	Note
20153074	OL336374.1	GII.4 Sydney[P16]	B
21161051	OL336377.1	GII.4 Sydney[P16]	B
21011037	OL336375.1	GII.4 Sydney[P16]	B
20062001	OL336383.1	GII.4 Sydney[P16]	B
20121007	OL336373.1	GII.4 Sydney[P16]	B
21121067	OL336376.1	GII.4 Sydney[P16]	B
21051018	OL336389.1	GII.4 Sydney[P16]	B
21161058	OL336380.1	GII.4 Sydney[P16]	B
20153072	OL336387.1	GII.4 Sydney[P16]	B
21063054	OL336385.1	GII.4 Sydney[P16]	B
unknown	ON329737.1	GII.4 Sydney[P16]	B
Jackson4273	MK762624.1	GII.4 Sydney[P16]	B
526-3	MT367328.1	GII.4 Sydney[P16]	B
782-2	MT344180.1	GII.4 Sydney[P16]	B
259-3	MT238672.1	GII.4 Sydney[P16]	B
259-2	MT238671.1	GII.4 Sydney[P16]	B
259-1	MT238670.1	GII.4 Sydney[P16]	B
782-3	MT238668.1	GII.4 Sydney[P16]	B
782-1	MT238667.1	GII.4 Sydney[P16]	B
259-2	MT537941.1	GII.4 Sydney[P16]	B
259-3	MT537634.1	GII.4 Sydney[P16]	B
259-2	MT537633.1	GII.4 Sydney[P16]	B
259-1	MT537605.1	GII.4 Sydney[P16]	B
CMH-N010-18	MW305723.1	GII.4 Sydney[P16]	B
Davidson0138	MT031821.1	GII.4 Sydney[P16]	B
Polk0185	MN897755.1	GII.4 Sydney[P16]	B
Kansas1621	OL898515.1	GII.4 Sydney[P16]	B
1815Y095	OL336335.1	GII.4 Sydney[P16]	B
1914Y048	OL336336.1	GII.4 Sydney[P16]	B
1914Y138	OL336337.1	GII.4 Sydney[P16]	B
2005Y0085	OL336338.1	GII.4 Sydney[P16]	B
2101Y105	OL336340.1	GII.4 Sydney[P16]	B
1717Y007	OL336332.1	GII.4 Sydney[P16]	B
2005Y0127	OL336339.1	GII.4 Sydney[P16]	B
2108Y2413	OL336341.1	GII.4 Sydney[P16]	B
BNE4	MG002633.1	GII.4 Sydney[P16]	B
BMH19-137	MW661283.1	GII.4 Sydney[P16]	B
Bay0244	MK764016.1	GII.4 Sydney[P16]	B
BNE2	MG002631.1	GII.4 Sydney[P16]	B
ETR-NV-268	MK762724.1	GII.4 Sydney[P16]	B
Nsk-N4740	KF920739.4	GII.P16-GII.16	B
Neustrelitz260	AY772730.1	GII.P16-GII.16	B
Asia	AB220921.1	GII.4 Asia	C
Sakai04179	AB220922.1	GII.4 Asia	C
Isumi060936	AB294790.1	GII.4 Yerseke	C
Osaka	AB434770.1	GII.4 Osaka	C
Hokkaido3	AB933767.1	GII.4 New Orleans	C
US95-96	AF414424.1	GII.4 US95-96	C
Langen1061	AY485642.1	GII.4 Farmington Hills	C
FarmingtonHills	AY502023.1	GII.4 Farmington Hills	C
Hunter504D04O	DQ078814.2	GII.4 Hunter	C
Yerseke	EF126963.1	GII.4 Yerseke	C
DenHaag	EF126965.1	GII.4 DenHaag	C
Minerva	EU078417.1	GII.4 DenHaag	C
PunePC51	EU921388.2	GII.4 Osaka	C
NSW001P	GQ845367.2	GII.4 New Orleans	C
CUHK3655	JX629458.1	GII.4 Sydney[P31]	C
2015-SP-0389_121215_MD	KX354141.1	GII.4 Sydney[P16]	C
2015-SP-0390_121715_MD	KX354142.1	GII.4 Sydney[P16]	C
2015-SP-0378_121415_CA	KX354143.1	GII.4 Sydney[P16]	C
2015-SP-0379_121415_CA	KX354144.1	GII.4 Sydney[P16]	C
2015-SP-0382_120515_IN	KX354145.1	GII.4 Sydney[P16]	C
2015-SP-0385_111315_LAC	KX354146.1	GII.4 Sydney[P16]	C
CA3477	KX907727.1	GII.4 Sydney[P16]	C
NS16-C38	KY210980.3	GII.4 Sydney[P16]	C
JC167	KY407088.1	GII.4 Sydney[P16]	C
JC176	KY407089.1	GII.4 Sydney[P16]	C
JC172	KY407090.1	GII.4 Sydney[P16]	C
JC215	KY407109.1	GII.4 Sydney[P16]	C

Strain name	GenBank accession numbers	Genotype	Note
JC232	KY407124.1	GII.4 Sydney[P16]	C
JC258	KY407160.1	GII.4 Sydney[P16]	C
JC263	KY407162.1	GII.4 Sydney[P16]	C
JC207	KY407164.1	GII.4 Sydney[P16]	C
JC275	KY407165.1	GII.4 Sydney[P16]	C
016O01	KY407207.1	GII.4 Sydney[P16]	C
016P01	KY407210.1	GII.4 Sydney[P16]	C
CUHK-NS-1283	KY679167.1	GII.4 Sydney[P16]	C
CUHK-NS-1297	KY679170.1	GII.4 Sydney[P16]	C
NOR-2559	KY887599.1	GII.4 Sydney[P16]	C
NOR-2558	KY887600.1	GII.4 Sydney[P16]	C
NOR-2565	KY887601.1	GII.4 Sydney[P16]	C
NOR-2516	KY887602.1	GII.4 Sydney[P16]	C
NOR-2518	KY887604.1	GII.4 Sydney[P16]	C
NOR-2520	KY887605.1	GII.4 Sydney[P16]	C
QLDB309	KY905335.1	GII.4 Sydney[P16]	C
CS4243	KY947549.1	GII.4 Sydney[P16]	C
Pasadena 3477	KY947550.1	GII.4 Sydney[P16]	C
OH16002	LC153121.1	GII.4 Sydney[P16]	C
OC16023	LC153122.1	GII.4 Sydney[P16]	C
AichiF104	LC325217.1	GII.4 Sydney[P16]	C
AichiF25	LC331997.1	GII.4 Sydney[P16]	C
Hu/GII.4[P16]/19-187/Osaka/2020/JPN	LC645996.1	GII.4 Sydney[P16]	C
BNE1	MG002630.1	GII.4 Sydney[P16]	C
BNE2	MG002631.1	GII.4 Sydney[P16]	C
NS17-A869	MG892929.3	GII.4 Sydney[P16]	C
NORO_227_07_01_2016	MH218685.1	GII.4 Sydney[P16]	C
GZ2016-L550	MH469188.1	GII.4 Sydney[P16]	C
AlbertaEI084	MH922873.1	GII.4 Sydney[P16]	C
AlbertaEI277	MH922874.1	GII.4 Sydney[P16]	C
AlbertaEI487	MH922875.1	GII.4 Sydney[P16]	C
AlbertaEI231	MH922876.1	GII.4 Sydney[P16]	C
Fresno-0182	MK073885.1	GII.4 Sydney[P16]	C
Roscommon-0105	MK073888.1	GII.4 Sydney[P16]	C
Gallatin-0036	MK073890.1	GII.4 Sydney[P16]	C
Gallatin-0038	MK073891.1	GII.4 Sydney[P16]	C
Nashville-0085	MK073894.1	GII.4 Sydney[P16]	C
MK213539.1	MK213539.1	GII.4 Sydney[P16]	C
NSW8653	MK213540.1	GII.4 Sydney[P16]	C
NSW3648	MK213541.1	GII.4 Sydney[P16]	C
NSW9639	MK213542.1	GII.4 Sydney[P16]	C
NSW17N128	MK213543.1	GII.4 Sydney[P16]	C
NSW6351	MK213544.1	GII.4 Sydney[P16]	C
NSW9983	MK213545.1	GII.4 Sydney[P16]	C
NSW17N100	MK213546.1	GII.4 Sydney[P16]	C
NSW3704	MK213547.1	GII.4 Sydney[P16]	C
NS18-A1494	MK483909.2	GII.4 Sydney[P16]	C
Auglaize0259	MK629457.1	GII.4 Sydney[P16]	C
Grant0016	MK752933.1	GII.4 Sydney[P16]	C
rant0015	MK752934.1	GII.4 Sydney[P16]	C
St.Croix0439	MK752936.1	GII.4 Sydney[P16]	C
CerroGordo0434	MK752937.1	GII.4 Sydney[P16]	C
Brown0441	MK752938.1	GII.4 Sydney[P16]	C
Marathon0626	MK752942.1	GII.4 Sydney[P16]	C
LaCrosse0643	MK752943.1	GII.4 Sydney[P16]	C
Lane0371	MK753017.1	GII.4 Sydney[P16]	C
Clackamas0373	MK753018.1	GII.4 Sydney[P16]	C
SanDiego0043	MK753029.1	GII.4 Sydney[P16]	C
Marin0375	MK753030.1	GII.4 Sydney[P16]	C
SanJoaquin0116	MK753032.1	GII.4 Sydney[P16]	C
BaltimoreCity0391	MK754442.1	GII.4 Sydney[P16]	C
Baltimore0201	MK754444.1	GII.4 Sydney[P16]	C
Washington0207	MK754446.1	GII.4 Sydney[P16]	C
Alameda0771	MK756033.1	GII.4 Sydney[P16]	C
Alameda0766	MK756034.1	GII.4 Sydney[P16]	C
Alameda0787	MK756035.1	GII.4 Sydney[P16]	C
Alameda0788	MK756036.1	GII.4 Sydney[P16]	C
Alameda0792	MK756037.1	GII.4 Sydney[P16]	C
Alameda0791	MK756038.1	GII.4 Sydney[P16]	C

Strain name	GenBank accession numbers	Genotype	Note
CS0193	MK762562.1	GII.4 Sydney[P16]	C
CS0194	MK762563.1	GII.4 Sydney[P16]	C
CS0195	MK762564.1	GII.4 Sydney[P16]	C
CS0275	MK762566.1	GII.4 Sydney[P16]	C
CS0276	MK762567.1	GII.4 Sydney[P16]	C
CS0651	MK762568.1	GII.4 Sydney[P16]	C
CS1009	MK762569.1	GII.4 Sydney[P16]	C
CS1010	MK762570.1	GII.4 Sydney[P16]	C
Jackson1608	MK762621.1	GII.4 Sydney[P16]	C
Jackson4273	MK762624.1	GII.4 Sydney[P16]	C
Lee0113	MK762629.1	GII.4 Sydney[P16]	C
Natrona0468	MK762632.1	GII.4 Sydney[P16]	C
LosAngeles0190	MK762635.1	GII.4 Sydney[P16]	C
LosAngeles0196	MK762636.1	GII.4 Sydney[P16]	C
LaPorte0381	MK762637.1	GII.4 Sydney[P16]	C
Miami0181	MK762638.1	GII.4 Sydney[P16]	C
Porter0184	MK762639.1	GII.4 Sydney[P16]	C
Hu/BT/2013/GII.P16-GII.4_Sydney2012/ETR-NV-268	MK762724.1	GII.4 Sydney[P16]	C
Hardin1028	MK762746.1	GII.4 Sydney[P16]	C
Wayne0078	MK764013.1	GII.4 Sydney[P16]	C
Ingham0228	MK764015.1	GII.4 Sydney[P16]	C
Bay0244	MK764016.1	GII.4 Sydney[P16]	C
Stafford0184	MK764018.1	GII.4 Sydney[P16]	C
Loudoun0411	MK764021.1	GII.4 Sydney[P16]	C
Olmsted0258	MK773584.1	GII.4 Sydney[P16]	C
Olmsted0261	MK773585.1	GII.4 Sydney[P16]	C
Ramsey0269	MK773586.1	GII.4 Sydney[P16]	C
Wood0352	MK775032.1	GII.4 Sydney[P16]	C
MN220720.1	MN220720.1	GII.4 Sydney[P16]	C
CUHK-NS-2200	MN400355.2	GII.4 Hong Kong	C
JH28	MN525275.1	GII.4 Sydney[P16]	C
JH29	MN525276.1	GII.4 Sydney[P16]	C
11617	MN782359.1	GII.4 Sydney[P16]	C
Polk0185	MN897755.1	GII.4 Sydney[P16]	C
Shawano0193	MN897756.1	GII.4 Sydney[P16]	C
SCD1055_1	MN996297.1	GII.4 Sydney[P16]	C
SCD1062_2	MN996299.1	GII.4 Sydney[P16]	C
MT029316.1	MT029316.1	GII.4 Sydney[P16]	C
Brevard0199	MT031761.1	GII.4 Sydney[P16]	C
Buchanan0126	MT032004.1	GII.4 Sydney[P16]	C
526-1	MT238666.1	GII.4 Sydney[P16]	C
782-1	MT238667.1	GII.4 Sydney[P16]	C
782-3	MT238668.1	GII.4 Sydney[P16]	C
259-1	MT238670.1	GII.4 Sydney[P16]	C
259-2	MT238671.1	GII.4 Sydney[P16]	C
259-3	MT238672.1	GII.4 Sydney[P16]	C
526-2	MT344179.1	GII.4 Sydney[P16]	C
782-2	MT344180.1	GII.4 Sydney[P16]	C
526-3	MT367328.1	GII.4 Sydney[P16]	C
259-1	MT537605.1	GII.4 Sydney[P16]	C
259-2	MT537633.1	GII.4 Sydney[P16]	C
259-3	MT537634.1	GII.4 Sydney[P16]	C
259-2	MT537941.1	GII.4 Sydney[P16]	C
55HBO	MW305548.1	GII.4 Sydney[P16]	C
170HBO	MW305553.1	GII.4 Sydney[P16]	C
211HBO	MW305557.1	GII.4 Sydney[P16]	C
249HBO	MW305558.1	GII.4 Sydney[P16]	C
278HBO	MW305560.1	GII.4 Sydney[P16]	C
CMH-N010-18	MW305723.1	GII.4 Sydney[P16]	C
B5073	MW521113.1	GII.4 Sydney[P16]	C
B7265	MW521126.1	GII.4 Sydney[P16]	C
B7446	MW521127.1	GII.4 Sydney[P16]	C
B7646	MW521129.1	GII.4 Sydney[P16]	C
B7826	MW521130.1	GII.4 Sydney[P16]	C
BMH16-074	MW661254.1	GII.4 Sydney[P16]	C
BMH18-085	MW661256.1	GII.4 Sydney[P16]	C
G4_11	MZ478136.1	GII.4 Sydney[P16]	C
G9_4.5	MZ478139.1	GII.4 Sydney[P16]	C

Strain name	GenBank accession numbers	Genotype	Note
G11_6	MZ478140.1	GII.4 Sydney[P16]	C
G19_10	MZ478141.1	GII.4 Sydney[P16]	C
Nizhny_Novgorod2275	MZ958411.1	GII.4 Sydney[P16]	C
NORO_226_06_01_2016	NC_039477.1	GII.4 Sydney[P16]	C
2105Y1677	OL336352.1	GII.4 Sydney[P16]	C
2105Y1888	OL336353.1	GII.4 Sydney[P16]	C
2105Y1889	OL336354.1	GII.4 Sydney[P16]	C
2105y1919	OL336355.1	GII.4 Sydney[P16]	C
2105Y1920	OL336356.1	GII.4 Sydney[P16]	C
2105Y1922	OL336357.1	GII.4 Sydney[P16]	C
20051042	OL336358.1	GII.4 Sydney[P16]	C
21011037	OL336359.1	GII.4 Sydney[P16]	C
20121007	OL336360.1	GII.4 Sydney[P16]	C
20153074	OL336361.1	GII.4 Sydney[P16]	C
2005Y130	OL336362.1	GII.4 Sydney[P16]	C
21121067	OL336363.1	GII.4 Sydney[P16]	C
1908Y813	OL336364.1	GII.4 Sydney[P16]	C
21161058	OL336365.1	GII.4 Sydney[P16]	C
21161051	OL336366.1	GII.4 Sydney[P16]	C
20161008	OL336367.1	GII.4 Sydney[P16]	C
19121200	OL336382.1	GII.4 Sydney[P16]	C
20062001	OL336383.1	GII.4 Sydney[P16]	C
20051046	OL336384.1	GII.4 Sydney[P16]	C
21063054	OL336385.1	GII.4 Sydney[P16]	C
1915Y216	OL336386.1	GII.4 Sydney[P16]	C
20153072	OL336387.1	GII.4 Sydney[P16]	C
21161049	OL336388.1	GII.4 Sydney[P16]	C
21051018	OL336389.1	GII.4 Sydney[P16]	C
Kansas11006	OL898512.1	GII.4 Sydney[P16]	C
Kansas0843	OL898514.1	GII.4 Sydney[P16]	C
Kansas1621	OL898515.1	GII.4 Sydney[P16]	C
Unknown	ON329737.1	GII.4 Sydney[P16]	C
Nizhny_Novgorod127	OP456985.1	GII.4 Sydney[P16]	C
Nizhny_Novgorod166	OP456986.1	GII.4 Sydney[P16]	C
Nizhny_Novgorod195	OP456987.1	GII.4 Sydney[P16]	C
Nizhny_Novgorod205	OP456988.1	GII.4 Sydney[P16]	C
Nizhny_Novgorod385	OP456989.1	GII.4 Sydney[P16]	C
Nizhny_Novgorod1269	OP456990.1	GII.4 Sydney[P16]	C
Nizhny_Novgorod1376	OP456991.1	GII.4 Sydney[P16]	C
Nizhny_Novgorod2782	OP456992.1	GII.4 Sydney[P16]	C
Nizhny_Novgorod2817	OP456993.1	GII.4 Sydney[P16]	C
Nizhny_Novgorod564	OP456994.1	GII.4 Sydney[P16]	C
Nizhny_Novgorod582	OP456995.1	GII.4 Sydney[P16]	C
Nizhny_Novgorod650	OP456996.1	GII.4 Sydney[P16]	C
Nizhny_Novgorod655	OP456997.1	GII.4 Sydney[P16]	C
Nizhny_Novgorod890	OP456998.1	GII.4 Sydney[P16]	C
Nizhny_Novgorod775	OP456999.1	GII.4 Sydney[P16]	C
Nizhny_Novgorod821	OP457000.1	GII.4 Sydney[P16]	C
Nizhny_Novgorod841	OP457001.1	GII.4 Sydney[P16]	C
Nizhny_Novgorod2005	OP457002.1	GII.4 Sydney[P16]	C
izhny_Novgorod2086	OP457003.1	GII.4 Sydney[P16]	C
Nizhny_Novgorod14	OP457004.1	GII.4 Sydney[P16]	C
Nizhny_Novgorod58	OP457005.1	GII.4 Sydney[P16]	C
Nizhny_Novgorod3370	OP518601.1	GII.4 Sydney[P16]	C
Nizhny_Novgorod567	OP712198.1	GII.4 Sydney[P16]	C

*A, B and C indicate genomic, RdRp and VP1 nucleotide sequences used for analysis, respectively.

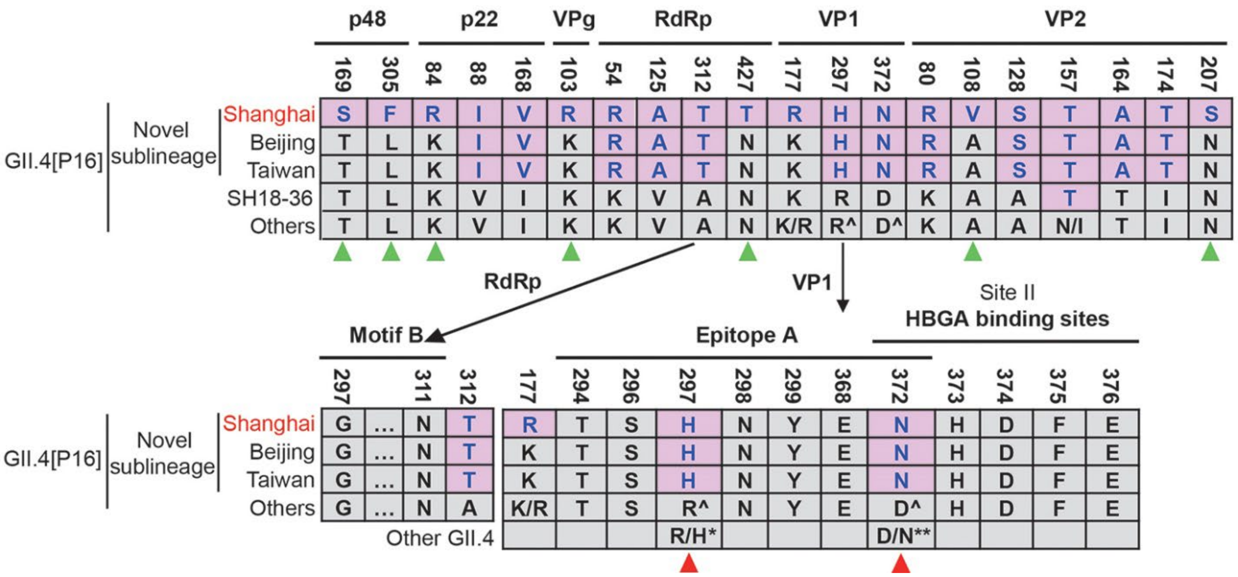

Appendix Figure 1. Sequence analysis of the newly identified GII.4 Sydney[P16] noroviruses in Shanghai, China, 2020-2022. List of unique amino acid substitutions in the nonstructural and structural proteins of Shanghai GII.4 Sydney[P16] strains identified in the study compared with other regional SHGII.4-2020 and other GII.4 Sydney[P16] strains available in GenBank. Strains in the GII.4 Sydney[P16] cluster indicate additional previous strains. Residues are numbered based on the sequence of SHGII.4-2020. Amino acid mutations in SHGII.4-2020 not found in other GII.4 Sydney[P16] sequences are highlighted in purple shading. Green triangles indicate the amino acid mutations found only in Shanghai GII.4 Sydney[P16] sequences obtained in our study, while other mutations indicated by purple shading were found in all sequences of SHGII.4-2020. Amino acid mutations in SHGII.4-2020 associated with epitope A and HBGA binding site II are denoted by red triangles. Two residues of 297 and 372 in VP1 were compared with all GII.4 noroviruses. ^Most previous GII.4 Sydney[P16] strains harbor R297 and D372 in VP1, but only certain strains (MG002631.1, MH922876, and MK754444) contain 297H and 372N in VP1; *other amino acids including S/Q/G/W/E/A/P/T/K; **other amino acids including S/E/Q/T/K/H/I/G/Y/V.

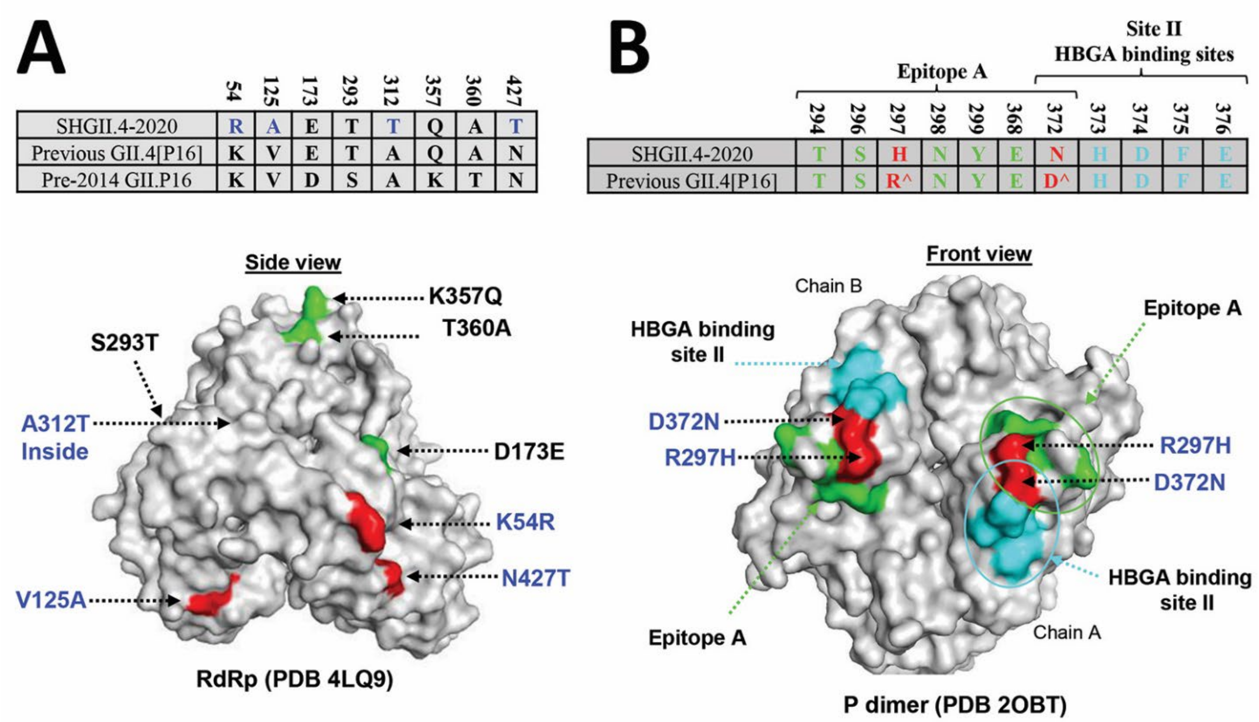

Appendix Figure 2. Structural mapping of the unique amino acid substitutions in the RNA-dependent RNA polymerase (RdRp) (A) and P domain (B) of SHGII.4-2020, mapped on the structural model of GII.P4 RdRp (PDB:4LQ9) and GII.4 P dimer (PDB: 2OBT). ^Most previous GII.4 Sydney[P16] strains harbor R297 and D372 in the VP1, but only certain strains (MG002631.1, MH922876, and MK754444) contain 297H and 372N. The newly identified amino acid substitutions in the RdRp and P domain of SHGII.4-2020 are indicated in red. The amino acid substitutions present in the RdRp of the previous GII.4 Sydney[P16] noroviruses are highlighted in green. The amino acids for the composition of epitope A and HBGA binding site II in the P domain are marked in cyan and green, and the mutant sites identified in SHGII.4-2020 are highlighted in red.

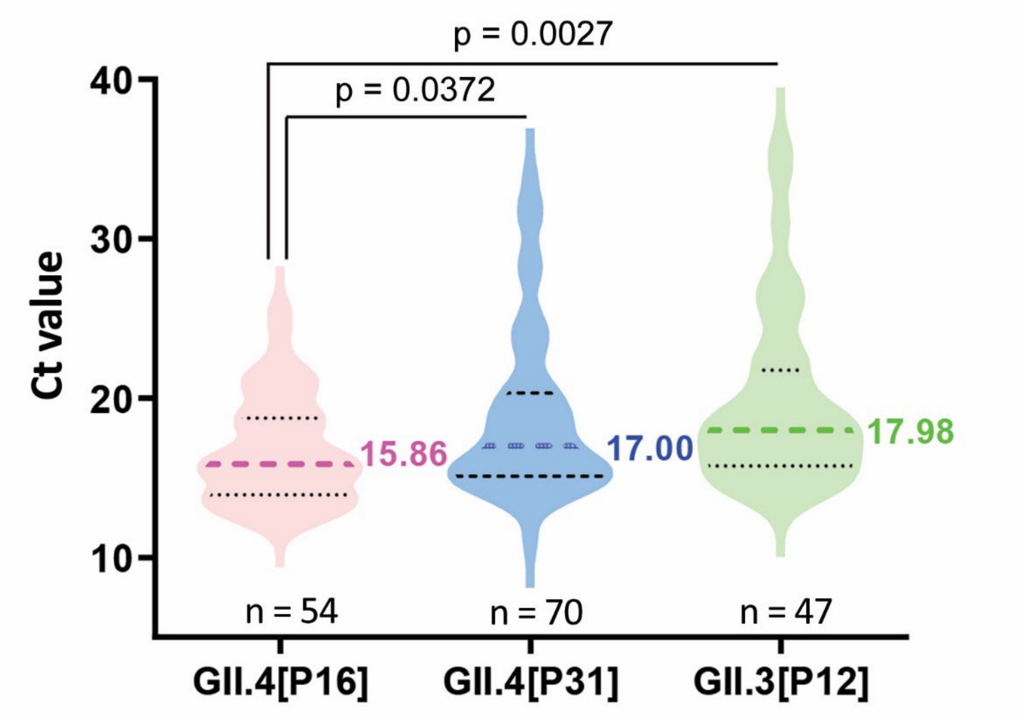

Appendix Figure 3. Comparison of viral loads (Ct values) in fecal samples from SHGII.4-2020, GII.4 Sydney[P31], and GII.3[P12] case-patients. A lower Ct value represents a higher norovirus load. Three dotted lines indicate medians (bold) and interquartile ranges. Comparison of 2 continuous variables was conducted using Mann-Whitney U-test. A two-tailed p value <0.05 was considered statistically significant.
